# Supplementary figures and images for: A tumor-suppressive role of the PRC1 Polycomb epigenetic complex in the maintenance of adult Drosophila intestinal stem cell identity
Source: PLoS Genet. 2026 Jul 17;22(7):e1012226. doi: 10.1371/journal.pgen.1012226 (PMC13399535; doi:10.1371/journal.pgen.1012226)

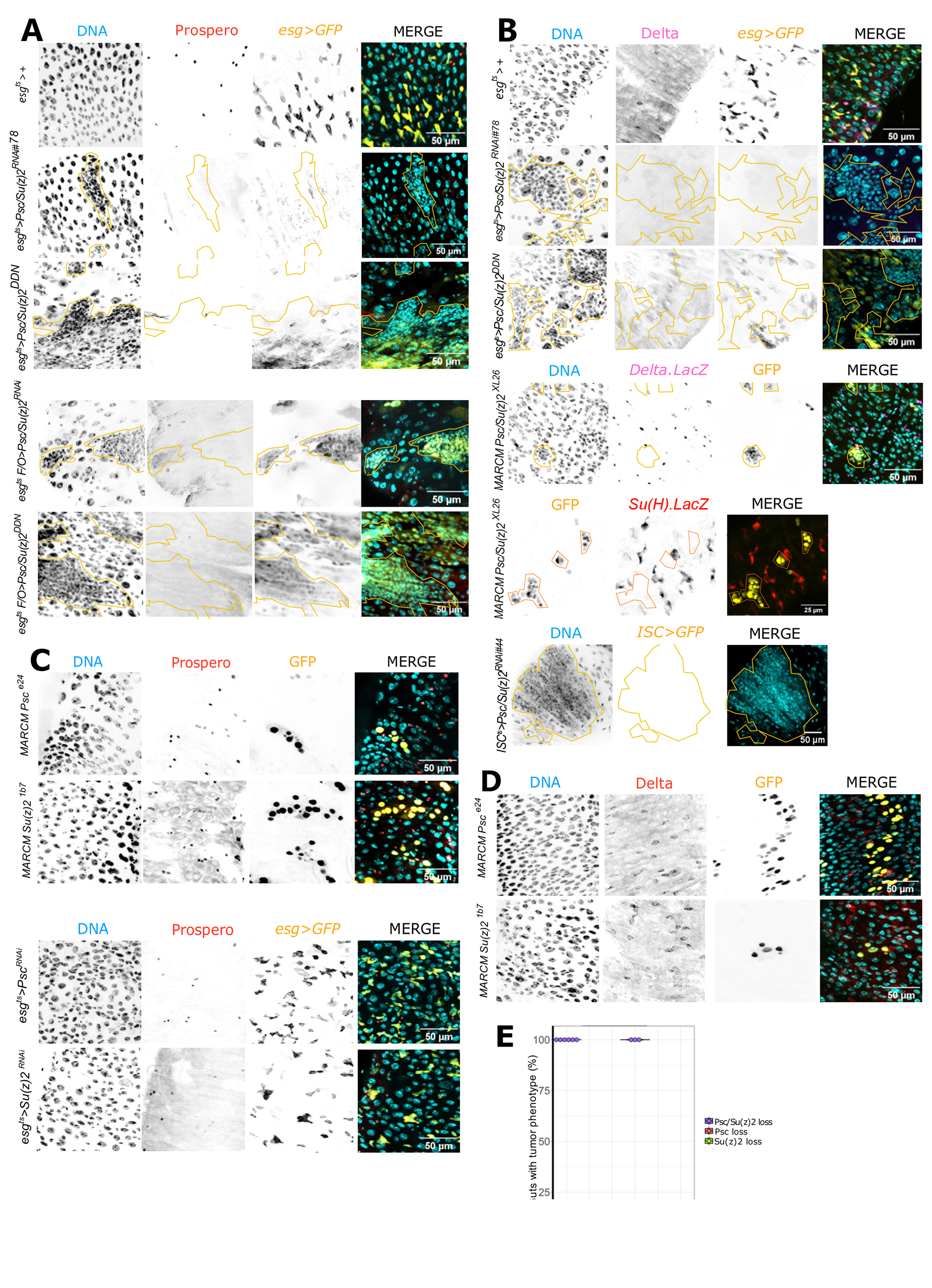

Supplement: S1 Fig — A. esg-positive cells expressing GFP (yellow) alone (control; upper panels) or together with Psc/Su(z)2RNAi#78 (second row panels) or double dominant negative forms of Psc and Su(z)2 (third row panels). esg-positive daughter cells (esgts F/O) expressing GFP together with Psc/Su(z)2RNAi#44 (used in main figures, fourth row panels) or double dominant negative forms of Psc and Su(z)2 (fifth row panels). The differentiated EE were labeled with the anti-Prospero antibody (red). B. Same three top panels as in D with the ISCs labeled with anti-Delta (Magenta). Psc/Su(z)2XL26 mutant MARCM clones expressing GFP (yellow) together with the ISC reporter gene Delta.LacZ (Magenta, fourth row panels). esg-Gal4ts,Su(H)-Gal80 (ISCts) expressing GFP (yellow) together with Psc/Su(z)2RNAi#44 (bottom panels). C. Psce24 (single Psc mutant; top panels) or Su(z)21b7 (single Su(z)2 mutant; second row panels) MARCM clones expressing GFP (yellow). esg-positive cells expressing GFP (yellow) together with PscRNAi (alone; third row panels) or Su(z)2RNAi (alone; bottom panels). The differentiated EE were marked with the anti-Prospero antibody (red). D. Psce24 (single Psc mutant; top panels) or Su(z)21b7 (single Su(z)2 mutant; second row panels) MARCM clones expressing GFP (yellow) with ISCs labeled with the anti-Delta antibody (red). E. Quantification of guts with tumor phenotype in %. Anova-Tukey multiple comparisons. One dot represents one replicate of n= 8-15 guts each. Tumors are outlined with yellow lines. DNA (turquoise): stained with DAPI. Scale bar, 50 μm. (TIF) [file pgen.1012226.s001.tif]

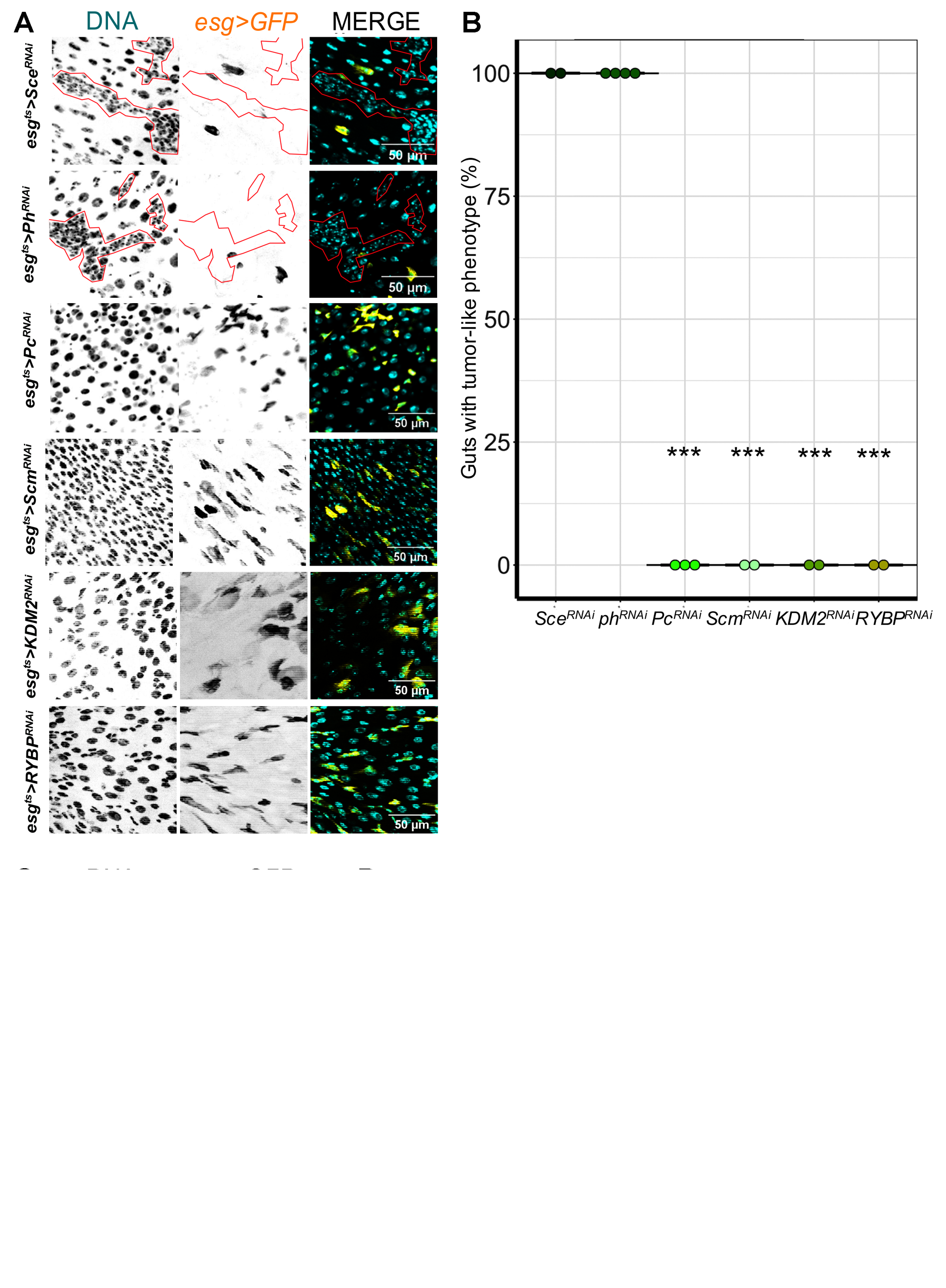

Supplement: S2 Fig — A. RNAi loss of function targeting the PRC1 subunits Sce, Ph, Pc, Scm, KDM2 and RYBP in esg-positive cells expressing GFP (yellow). B. Quantification of guts with tumor phenotype in %. C. esg-driven expression of GFP and Psc/Su(z)2RNAi for 14d continuously or transiently for 24 or 48h followed by a period of 13 days or 12 days, respectively, before dissection (to reach a total of 14d). D. Quantification of the number of cells per tumor in each condition. Anova-Tukey multiple comparisons. One dot represents one replicate of n= 8-12 guts each. Tumors are outlined with red lines. DNA: stained with DAPI. Scale bar, 50 μm or 100 μm as indicated. (TIF) [file pgen.1012226.s002.tif]

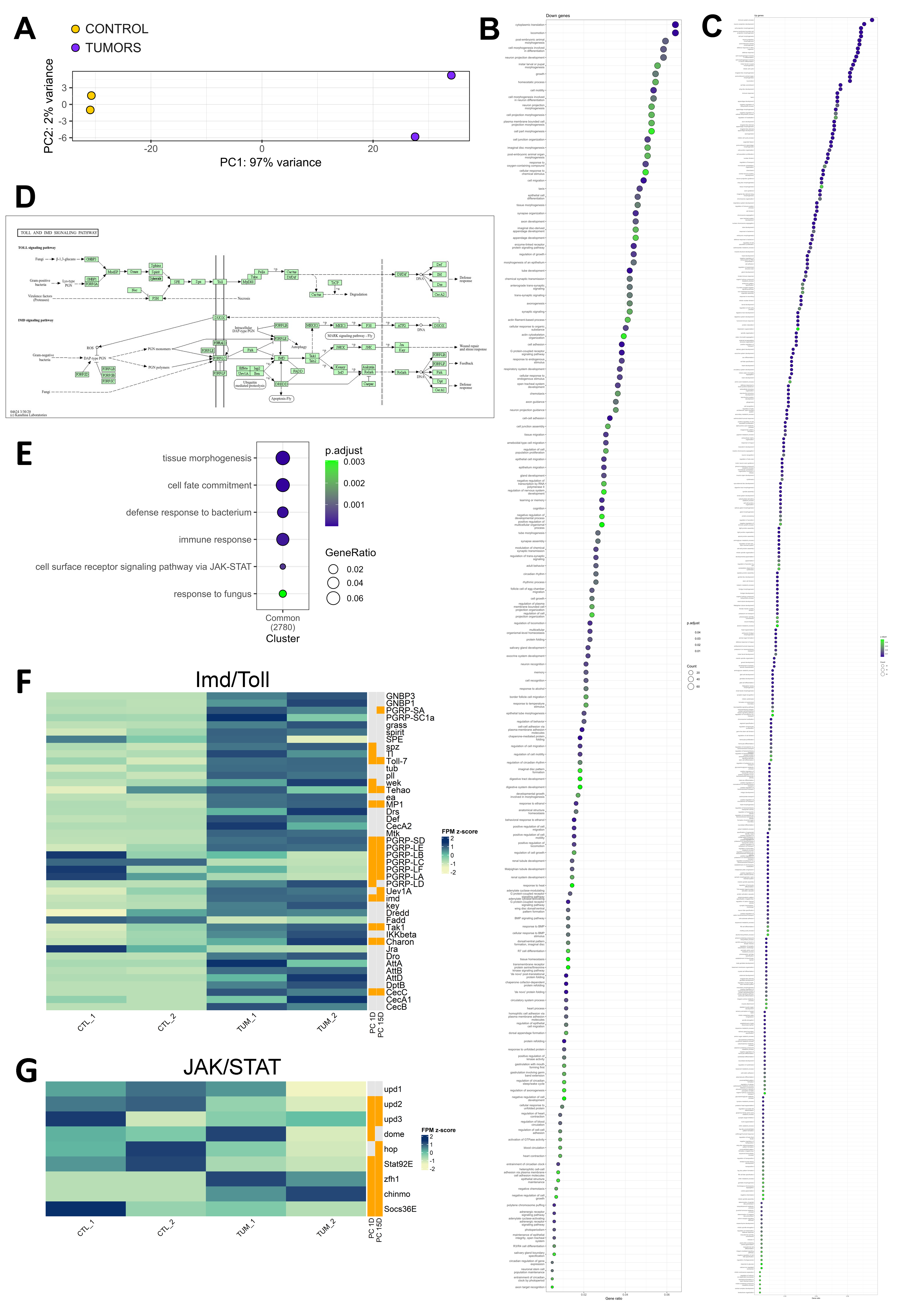

Supplement: S3 Fig — A. Principal component analysis (PCA) of normalized RNA-seq read counts for tumor and control conditions. Each dot corresponds to one biological replicate. Similarity between samples from one condition is reflected in their close distance. B and C. Gene Ontology terms enriched in downregulated (B) and upregulated (C) genes of the RNA sequencing data. D. Toll and Imd pathways in Drosophila, from flybase data. E. Selected Gene Ontology terms enriched in the Pc-profiling CUT&Tag data recently published [45], after our own re-analysis. F. Genes of the Toll/Imd pathways that are direct targets of Pc (CUT&Tag; in orange) in association with the heatmap of their transcriptional regulation in TIIC tumors (RNA sequencing). G. Genes of the JAK/STAT pathway that are direct targets of Pc (CUT&Tag; in orange) in association with the heatmap of their transcriptional regulation in TIIC tumors (RNA sequencing). 1D = 1 day-old flies (young); 15D = 15 day-old flies (middle-aged). (TIF) [file pgen.1012226.s003.tif]

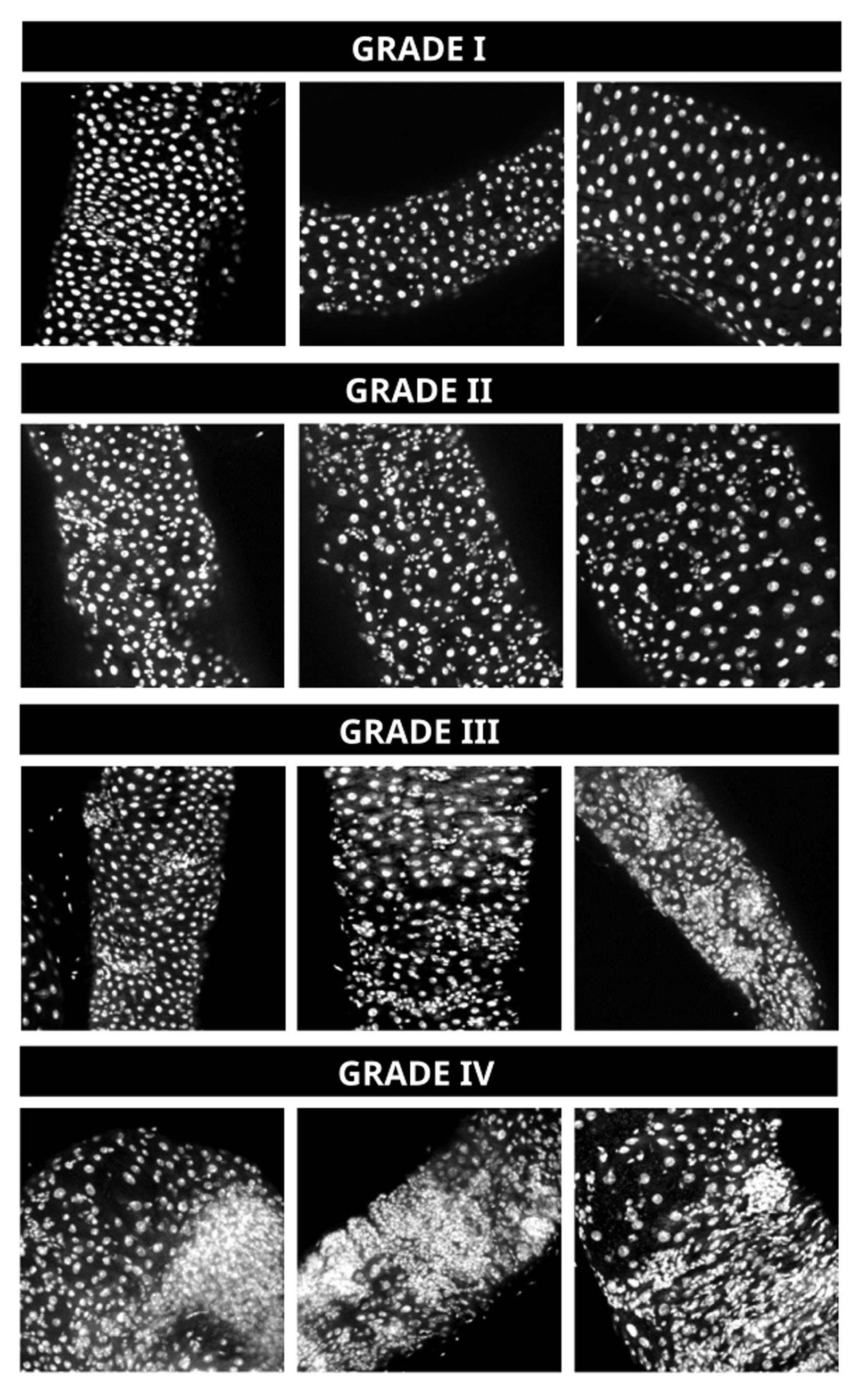

Supplement: S4 Fig — Following the table below, tumor burden was determined by analyzing images in FIJI, with image names concealed to ensure a blind assessment. Tumor grades were classified as follows: Grade I: rare clusters of few cells; Grade II: several clusters of few cells; Grade III: medium size clusters; Grade IV: massive clusters. (S4_Fig.TIF) [file pgen.1012226.s004.tif]
