# Supplementary material for: A tumor-suppressive role of the PRC1 Polycomb epigenetic complex in the maintenance of adult Drosophila intestinal stem cell identity
Source: PLoS Genet. 2026 Jul 17;22(7):e1012226. doi: 10.1371/journal.pgen.1012226 (PMC13399535; doi:10.1371/journal.pgen.1012226)
Supplement: Attachment — Submitted filename: Joly_PRC1 tumor_Plos G 2025.docx [file pgen.1012226.s009.docx]

In this manuscript, Joly, Rousset, and colleagues report that the Polycomb Repressive Complex 1 (PRC1) controls the proliferation and identity of adult intestinal stem cells (ISCs). Loss of PRC1 complex function led to loss of ISC identity and the transformation of ISCs into tumor-initiating intestinal cells (TIICs). The analysis is fairly extensive, and many of the findings are novel and interesting. Much of the data support the authors’ conclusions, and the quality of the data is good. One strength of the paper is the identification of PRC1’s function in controlling JAK-STAT signaling activation in ISCs. This broadens our understanding of the PRC1 complex’s functions, although the mechanism remains unclear. Another strength is that the activation of TOLL and IMD pathways in *PRC1-LOF* tumors plays an anti-tumor effect independent of cell death.

Overall, this manuscript is well-written, and the mechanistic study is logically organized. It will be a good fit for *PLOS Genetics* after a revision. Some specific comments are detailed below.

**Major:**

1.	As mentioned above, it’s quite interesting that *PRC1-LOF* could activate the JAK-STAT pathway in ISCs. However, it’s unclear whether this regulation is direct or indirect. There could be two potential mechanisms: (1) because of PRC1’s epigenetic repressive role, *PRC1-LOF* may increase STAT92E’s accessibility to its target genes. (2) *PRC1-LOF* increases ISC proliferation independent of JAK-STAT, but hyper-proliferated ISCs squeeze and stress the surrounding ECs. Stressed ECs then generate Upd3 cytokine to trigger JAK-STAT signaling in ISCs as a feedback regulation. This second indirect regulation has often been observed in fly gut tumor models (e.g., *Notch^RNAi^* tumors, *Yki^3SA^* tumors, *APC^-/-^* tumors, etc.).

To test the first mechanism, a Cut&Tag assay for STAT92E (w/ or w/o *Psc/Su(z)2^RNAi^*) may be required. This tremendous amount of work may be beyond the authors’ scope. But it is easy to test the second mechanism. The authors could use the *upd3-lacZ* reporter to demonstrate whether *PRC1-LOF* in ISCs could induce *upd3-lacZ* in ECs (*esg^ts^,UAS-GFP; upd3-lacZ>Psc/Su(z)2^RNAi^*). Furthermore, whether Upd2/3 mutants could diminish *Psc/Su(z)2^RNAi^*-caused ISC proliferation/tumor. To strengthen the model of this paper, I strongly encourage the authors to test the second mechanism.

2.	Since the *PRC1-LOF* tumor flies (*esg^ts^>Psc/Su(z)2^RNAi^*) do not have a significantly shortened lifespan compared to the control (Fig. 2E). I’m wondering whether the tumors in the *esg^ts^>Psc/Su(z)2^RNAi^* flies persist and continue to grow throughout the flies’ lifetimes, or whether they will eventually be diminished after shifting the flies back to 18 ℃.

3.	It's quite interesting that *PRC1-LOF* could decrease esg’s expression (Figs. 1D and S1A). However, using the *esg^ts^>UAS-GFP* in this assay is less compelling. I suggest using the *Miranda-Gal4* (another ISC/EB driver) to repeat this experiment by assaying *esg-lacZ* or *esg* qPCR. Similarly, in Figs. 1J-K, using *piezo-Gal4* is problematic, as it has some overlapping expression patterns with Dl-Gal4. Please repeat this experiment using the classic EE-driver *ProsV1-Gal4* or even *Rab3-Gal4*.

**Minor:**

1.	Fig. 2 needs supplementary data to show if the *esgF/O>Psc/Su(z)2^RNAi^* flies exhibited the tumor phenotype.

2.	Fig. 4A-B need the *Dorsal^RNAi^* and *Relish^RNAi^* alone controls.

3.	Page 14, lines 343-344, the authors missed a conclusion sentence for these data. Are Zfh1 and Chimno not required for the *PRC1^LOF^*-tumor progression?
