# Supplementary material for: A tumor-suppressive role of the PRC1 Polycomb epigenetic complex in the maintenance of adult Drosophila intestinal stem cell identity
Source: PLoS Genet. 2026 Jul 17;22(7):e1012226. doi: 10.1371/journal.pgen.1012226 (PMC13399535; doi:10.1371/journal.pgen.1012226)
Supplement: Attachment — Submitted filename: Rebuttal Letter_PLOS Genetics revision.docx [file pgen.1012226.s011.docx]

**Reviewer #1:**

*In this manuscript, Joly et al characterise the phenotype of loss-of-function of members of the polycomb repressive complex 1 (PRC1) in the Drosophila intestine. They show that loss of PRC1 results in clusters of cells that lose intestinal markers and become neoplastic. They also perform particularly impressive serial transplant experiments to show that these cells acquire the capacity to become malignant and metastatic. This study will be of interest to understanding how epigenetic regulators can influence cancer onset. There are a few issues that would improve the manuscript and help it achieve its fullest impact:*

***Reviewer 1 – Comment 1:***

*The characterisation of the PRC1-deficient cells could be expanded. While the authors show loss of esg expression and characterise proliferation, they claim that the cells lose intestinal identity. Indeed, this is in the title of the manuscript. It would be important to use several markers of intestinal and intestinal stem cell identity to support this statement, as loss of a single marker is not enough to draw strong conclusions about the identity of a cell type. Similarly, the RNAseq shows decreased expression of epithelial markers, but these are not validated in the tumours, despite the availability of many antibodies from the DSHB.*

We thank Reviewer 1 for this important comment, as stem cell identity loss is indeed a critical claim of our work. Our conclusion is based on the comprehensive loss of ISC markers in TIIC tumors. In the adult *Drosophila* midgut, Dl and Esg represent the two most robust and widely accepted markers of ISC identity. Dl was established as the definitive ISC marker by Ohlstein & Spradling (2007) and remains the gold standard in lineage tracing and turnover studies. Esg is essential for ISC maintenance (Korzelius et al., 2014; Loza-Coll et al., 2014), and loss of *esg* function rapidly eliminates Dl⁺ ISCs. Notably, while *esg* is also expressed in enteroblasts (EBs) and enteroendocrine precursors (EEPs), Dl expression is highly specific to ISCs, showing high levels of expression exclusively in these cells.

To strengthen our conclusions, we have expanded our analysis in several ways:

1. **RNAseq analysis:** We added *Delta* to the heatmap (Fig. 3B) along with *esg* and included additional validated ISC markers:

-	mira – Bardin et al., Development 2010

-	insc – Pandey & Roy, Experimental Cell Research 2024

-	sox21a – Doupé et al., PNAS 2018

-	zfh2 – Doupé et al., PNAS 2018

All of these markers show diminished expression in PRC1-deficient cells, consistent with loss of stem cell identity.

2. **Epithelial marker analysis:** We performed immunostaining for Armadillo/β-Catenin, a marker enriched at adherens junctions in ISC (Chen J et al. 2018 PLoS Biol 16(10): e3000041; Chen and Johnston, 2022 Frontiers in Cell and Developmental Biology, Volume 10 - Article 886773) found downregulated in the RNA sequencing data (Fig. 3B). Our new results indicate a decreased expression in tumor cells, further supporting the loss of intestinal epithelial characteristics (new Fig. 3D, E). Additionally, we attempted to analyze Shg/E-Cadherin expression, but technical limitations with the antibody prevented us from obtaining reliable results under our experimental conditions.
3. **Validation:** Our conclusion is supported by convergent evidence from three independent approaches: (i) RNA-seq transcriptomic profiling, (ii) reporter gene expression (*esg*-GFP, *Dl*-lacZ), and (iii) immunofluorescence with antibodies against Delta and Armadillo.

Importantly, PRC1-deficient cells also lack markers of differentiated lineages: they are negative for Prospero (enteroendocrine cells), and they do not display the large polyploid nuclear morphology characteristic of mature enterocytes. To be sure that they are not EB, we performed an additional experiment to test the expression of Su(H), which is specifically expressed in enteroblasts (Tian and Jiang. eLife 2014;3:e01857). The results showed no expression of the reporter gene Su(H)-lacZ in tumors, confirming that TIICs are not EB (results added in the existing Fig. S1B: new fifth row).

Together, the combined loss of ISC markers (Esg, Dl, Mira, Insc, Sox21a, Zfh2) and of epithelial markers (Armadillo), the absence of differentiation markers (Pros, Su(H)), the lack of terminal enterocyte morphology (big nuclei), and the comprehensive transcriptomic data converge on the interpretation that TIICs lose ISC and epithelial identities without adopting any specific differentiated lineage.

We have revised the text in the manuscript (marked-up copy) to clarify these critical points: for the ISC and epithelial markers, see lines 286-288 and lines 307-310; and for Su(H): see lines 165-168. We have also included appropriate citations to the foundational literature establishing these markers.

***Reviewer 1 – Comment 2:***

*Secondly, some of the RNAseq findings should be validated. The authors make several claims based only on the analysis of the sequencing data, but only show that JAK/STAT signalling is activated using the 10X-STAT-GFP reporter. This would be especially helpful when arguing that the Toll and Imd pathways are activated autonomously in the tumours, but showing upregulation of Zfh1 and Chinmo would also be useful. This would increase confidence in the RNAseq dataset and the authors’ conclusions. Similarly, when discussing the upregulation of JAK/STAT independently of the ligands, it would be important to validate that ligand expression is indeed unchanged, or use more caution when discussing the results (eg p.18, lines 448-9).*

We agree with Reviewer 1's comment on conclusions about gene or pathway involvement based solely on sequencing analysis. We would like to clarify that our study includes validation of key gene expression changes. Our RNA-seq data showing the downregulation of both *Psc* and *Su(z)2* (Fig. 3B) is consistent with our experimental design since these genes are the targets of our RNAi approach. Importantly, we validated critical gene expression changes using redundant methods: loss of *Delta* and *esg* expression was confirmed by immunostaining and reporter gene analysis (see Comment 1). In addition, we performed new immunostainings to validate the downregulation of epithelial marker expression (see also Comment 1).

**JAK/STAT pathway activation:**

We detected JAK/STAT activation using the widely accepted 10XSTAT-GFP reporter and importantly functionally validated its requirement for tumor growth through genetic manipulation. Regarding ligand expression, our RNA-seq data did not reveal significant upregulation of *upd1*, *upd2*, or *upd3* in the tumor cells themselves. To directly address whether ligand expression is indeed unchanged, we have now analyzed *upd3-lacZ* expression in guts bearing tumors (*esg^ts^,GFP,upd3-lacZ>Psc-Su(z)2^DDN^*; see also Reviewer 2 – Comment 1). The results indicate no expression of *upd3* in TIICs, confirming the RNA-seq data. We revised the manuscript to add this experiment (new Fig. 5C and lines 382-389).

Although our RNA-seq data indicates the upregulation of *chinmo* and *zfh1* in TIIC tumors, we have not independently validated these changes at the RNA or protein level. However, functional analyses using RNAi targeting these genes were unsuccessful (see Comment 3), reducing the priority for further validation of their expression levels.

**Toll and Imd pathway activation:**

Our functional experiments strongly support the activation of these pathways in tumor cells. Knockdown of *Dorsal* or *Relish* in TIICs significantly increased tumor size and mitotic activity, which is consistent with the notion that cell-autonomous activation of the Toll and Imd pathways restricts tumor expansion. To validate pathway activity directly, we initially tested the *Diptericin A(DptA)-cherry* reporter of Imd signaling but we observed no activation (data not shown). Re-examination of our RNA-seq data revealed that *DptB* (log2FC = 8.85), but not *DptA* (log2FC = 0.17), is strongly upregulated, explaining the negative result with the *DptA* reporter. Unfortunately, to our knowledge, no reporter exists for *DptB*. As an alternative approach to demonstrate pathway activation, we analyzed the *Drosomycin(Drs)-GFP* reporter of Toll signaling (*esg^ts^,Drs-GFP>Psc-Su(z)2^DDN^*). Notably, the *Drs* gene is substantially upregulated in our RNA-seq data (log2FC = 5.8), supporting strong activation of the Toll pathway. However, we were unable to observe activation of the *Drs-GFP* reporter in the TIIC tumors (see Figure Drs-GFP below). This construct corresponds to a transgene containing 2450bp of the *Drs* promoter, which is known to respond to immune signals (Ferrandon et al., 1998 EMBO Journal). This short promoter may omit sequences regulated by the loss of PRC1, which could explain the lack of activation in TIIC tumors.

**Figure Drs-GFP:** no expression of *Drs-GFP* in TIIC tumors (*esg^ts^,Drs-GFP>Psc-Su(z)2^DDN^*). The tumor (bottom panels) is outlined with the yellow line. *Drs-GFP* is normally not expressed in control intestinal cells (*esg^ts^,Drs-GFP>+*; top panels). DNA (turquoise): stained with DAPI. Scale bar, 50 μm.

To go further with this important question, we reanalyzed a recently published dataset for which the authors performed a CUT&Tag against the Polycomb Pc protein in the gut during aging (Leichter et al., 2026, Genome Res. gr.281058.125). Our new analysis of these published data revealed an enrichment of the GO terms “tissue morphogenesis” and “cell fate commitment”, as expected. Interestingly, there is also enrichment of “defense response to bacterium”, “immune response” and “response to fungus”, suggesting a direct binding of PRC1 to genes involved in immune response. In addition, some of the genes that we found upregulated in TIIC tumors by RNA sequencing are also direct targets of Pc in the gut, such as the ligand Spatzle (Spz) and the receptor Toll of the Toll pathway. These data support a hypothesis that the upregulation of the Toll/Imd pathways in TIIC tumors is a direct effect of the PRC1 loss. We have now added this analysis to the manuscript (Fig S3E, F and lines 319-323).

In summary, we validated our key RNA-seq findings through several approaches including immunostaining and reporter gene analysis. Most importantly, we conducted functional genetic experiments that provided strong support for our conclusions about pathway (JAK/STAT and NF-κB Toll/Imd) involvement in tumor development.

***Reviewer 1 – Comment 3:***

*The authors should be cautious when interpreting negative data from RNAi experiments. To conclude that Zfh1 and Chinmo are indeed not relevant to the PRC1 phenotype, it would be important to demonstrate that the two proteins are indeed knocked down by RNAi expression.*

Regarding the *chinmo* RNAi experiment, we used two established RNAi lines from the Bloomington Drosophila Stock Center: RRID:BDSC_26777 and RRID:BDSC_33638. Both have been validated as effective in reducing Chinmo expression (for instance: Chafino et al. eLife 2023;12:e84648 for #26777; Grmai et al., 2021, G3, 2021, 11(4), jkab067 - Dillard C et al., 2025, PLoS Biol 23(4): e3002663 for #33638). Regarding *zfh1*, we employed the RNAi line RRID:BDSC_29347, which has been functionally validated in previous studies (Zacharioudaki et al., *eLife* 2019, 8:e41637 - Boukhatmi and Bray. eLife 2018;7:e35954).

While these lines are robust in other contexts, we acknowledge the reviewer’s concern and attempted to validate their efficacy in our system. We performed immunofluorescence experiments to assess *zfh1* knockdown in the gut using the *esgF/O* driver and the RNAi line RRID:BDSC_29347 (*esgF/O>zfh^i#29347^*) (see Figure Zfh1 below). Surprisingly, we observed no reduction in *zfh1* expression, even when using the *zfh1* RNAi line VDRC_103205 (*esgF/O>zfh1^i#103205^*), which has been shown to be effective in the eye disc (Parreno et al., Nature 2024) (see Figure Zfh1 below). Regarding *chinmo*, we have not been able to acquire the antibody necessary to perform the experiment.

Given our inability to confirm RNAi-mediated knockdown of *zfh1* and *chinmo* in the gut and therefore lacking convenient tools to study their role, we have removed these data from the manuscript (lines 374-377 and 494-498). These results are not central to our main conclusions, and we appreciate the reviewer’s guidance in ensuring the robustness of our interpretations.

**Figure Zfh1:** no loss of Zfh1 expression in the gut upon RNAi using RRID:BDSC_29347 (top panels) or VDRC_103205 (bottom panels) (*esgF/O>zfh^i#29347^* or *zfh1^i#103205^*). The flip-out clones (*esg>GFP* in yellow) are outlined with the yellow dotted lines. Arrows indicate nuclear Zfh1 immunostaining (red) inside de flip-out clones (yellow arrows) or outside (white arrows). DNA (turquoise): stained with DAPI. Scale bar, 50 μm.

***Reviewer 1 – Minor comment:***

*A typo in the legend for Fig. 3 “transcriptomique” should be “transcriptomic”.*

We thank the reviewer for his/her careful reading of our manuscript. We have now corrected this typographical error in the Figure 3 legend.

**Reviewer #2:**

*In this manuscript, Joly, Rousset, and colleagues report that the Polycomb Repressive Complex 1 (PRC1) controls the proliferation and identity of adult intestinal stem cells (ISCs). Loss of PRC1 complex function led to loss of ISC identity and the transformation of ISCs into tumor-initiating intestinal cells (TIICs). The analysis is fairly extensive, and many of the findings are novel and interesting. Much of the data support the authors’ conclusions, and the quality of the data is good. One strength of the paper is the identification of PRC1’s function in controlling JAK-STAT signaling activation in ISCs. This broadens our understanding of the PRC1 complex’s functions, although the mechanism remains unclear. Another strength is that the activation of TOLL and IMD pathways in PRC1-LOF tumors plays an anti-tumor effect independent of cell death.*

*Overall, this manuscript is well-written, and the mechanistic study is logically organized. It will be a good fit for PLOS Genetics after a revision. Some specific comments are detailed below.*

***Reviewer 2 – Comment 1*:**

*As mentioned above, it’s quite interesting that PRC1-LOF could activate the JAK-STAT pathway in ISCs. However, it’s unclear whether this regulation is direct or indirect. There could be two potential mechanisms: (1) because of PRC1’s epigenetic repressive role, PRC1-LOF may increase STAT92E’s accessibility to its target genes. (2) PRC1-LOF increases ISC proliferation independent of JAK-STAT, but hyper-proliferated ISCs squeeze and stress the surrounding ECs. Stressed ECs then generate Upd3 cytokine to trigger JAK-STAT signaling in ISCs as a feedback regulation. This second indirect regulation has often been observed in fly gut tumor models (e.g., Notch^RNAi^ tumors, Yki^3SA^ tumors, APC^-/-^ tumors, etc.). To test the first mechanism, a Cut&Tag assay for STAT92E (w/ or w/o Psc/Su(z)2^RNAi^) may be required. This tremendous amount of work may be beyond the authors’ scope. But it is easy to test the second mechanism. The authors could use the upd3-lacZ reporter to demonstrate whether PRC1-LOF in ISCs could induce upd3-lacZ in ECs (esg^ts^,UAS-GFP; upd3-lacZ>Psc/Su(z)2^RNAi^). Furthermore, whether Upd2/3 mutants could diminish Psc/Su(z)2^RNAi^-caused ISC proliferation/tumor. To strengthen the model of this paper, I strongly encourage the authors to test the second mechanism.*

We are grateful to Reviewer 2 for this insightful comment. Our data demonstrate robust activation of JAK/STAT activity in TIICs as measured by the 10XSTAT-GFP reporter, and the functional requirement of this pathway for tumor growth. While our RNA-seq data did not detect the expression of JAK/STAT ligands (*upd1*, *upd2* and *upd3*) in PRC1-deficient tumor cells, we acknowledge that ligand production could contribute to pathway activation, as discussed with Reviewer 1 (see Comment 2). Distinguishing between the direct activation of JAK/STAT target genes by PRC1 loss through increased accessibility of STAT92E and the indirect feedback-mediated activation of Upd ligands represents an important question.

As mentioned by Reviewer 2, the CUT&Tag assay for STAT92E goes beyond the scope of this manuscript. However, we took advantage of the recently published CUT&Tag data analyzing the profile of the Pc protein in intestinal cells (Leichter et al., 2026, Genome Res. gr.281058.125) to estimate whether the JAK/STAT genes that are regulated in our RNA sequencing dataset could be potential direct targets of the Pc-containing PRC1 complexes. This-re-analysis suggests that most of these JAK/STAT genes (except *upd1*) can be directly regulated by PRC1 (new Fig. S3G). To test the proposed second mechanism (indirect JAK/STAT regulation by the ligands), we performed the suggested experiment to detect the *udp3* expression in cells surrounding the tumors (*esg^ts^,GFP;upd3-lacZ>Psc/Su(z)2^DDN^*; see also Reviewer 1 – Comment 2). We did not observe a change in expression neither inside the tumors, nor in the adjacent tissues, compared to control guts (new Fig. 5C). This result indicates that the second, indirect mechanism linked to stressed EC expressing JAK/STAT ligands is not involved.

We have modified the manuscript to add the re-analysis of the CUT&Tag for Pc and the *upd3-LacZ* experiment (lines 377-389).

***Reviewer 2 – Comment 2*:**

*Since the PRC1-LOF tumor flies (esg^ts^>Psc/Su(z)2^RNAi^) do not have a significantly shortened lifespan compared to the control (Fig. 2E). I’m wondering whether the tumors in the esg^ts^>Psc/Su(z)2^RNAi^ flies persist and continue to grow throughout the flies’ lifetimes, or whether they will eventually be diminished after shifting the flies back to 18 ℃.*

To determine whether tumors persist and continue to grow throughout the flies' lifespan, we quantified tumor growth at 14 and 28 days post-induction. Tumors were indeed maintained at 28 days; however, their size did not increase significantly between the two time points (see Figure 14d-28d below). This slow growth over time (maybe due to the negative regulation by the NF-κB Toll/Imd pathways) likely accounts for the absence of a decrease in lifespan observed under non-stress conditions.

**Figure 14d-28d:** Quantification of the number of cells per tumor (*esg^ts^>Psc/Su(z)2^RNAi^*) at 14 and 28 days post-induction. *Anova-Tukey multiple comparisons*.

Reviewer 2 suggested shifting the *esg^ts^>Psc/Su(z)2^RNAi^* flies back to 18°C to halt dsRNA production and determine whether this would lead to tumor regression. A previous study demonstrated that transient depletion of PRC1 subunits in the larval eye imaginal disc is sufficient to induce neoplastic transformation (Parreno et al., 2024 Nature 629). To check whether this also occurs in the gut, we performed a similar experiment in the adult *Drosophila* intestine: we transiently depleted the Psc/Su(z)2 subunits in adult intestinal progenitors for 24 or 48 hours (*esg^ts^>Psc/Su(z)2^RNAi^*), and switched back the flies to 18℃ for the remaining 12/13 days of the experiment. We observed that a transient loss of Psc/Su(z)2 for 24 or 48 hours was sufficient to induce the formation of TIIC tumors, with the 48-hour condition achieving an efficacy close to that of continuous 14-day depletion.

This result is particularly notable, showing that, reminiscent of the observations made in the larval eye imaginal disc, a transient loss of the epigenetic modulator PcG genes is also sufficient to induce neoplasia in adult tissue and that epigenetic alterations can drive adult cell reprogramming. We have therefore incorporated this experiment into the manuscript by revising the text and figures accordingly (new Fig. S2C, D; lines 265-275).

***Reviewer 2 – Comment 3*:**

*It's quite interesting that PRC1-LOF could decrease esg’s expression (Figs. 1D and S1A). However, using the esg^ts^>UAS-GFP in this assay is less compelling. I suggest using the Miranda-Gal4 (another ISC/EB driver) to repeat this experiment by assaying esg-lacZ or esg qPCR. Similarly, in Figs. 1J-K, using piezo-Gal4 is problematic,* *as it has some overlapping expression patterns with Dl-Gal4. Please repeat this experiment using the classic EE-driver ProsV1-Gal4 or even Rab3-Gal4.*

As for Reviewer 1 (see Comment 1), we thank Reviewer 2 for raising this important point. Our RNA-seq analysis independently confirms a strong loss of *esg* transcription in PRC1-deficient tumor cells (8.9-fold decrease; log₂FC = -3.15), fully consistent with the loss of GFP observed using the *esg^ts^* > GFP reporter. We also used the alternative FLP-FRT system: the *esgF/O* line, which allows cells to permanently express the transgenes (e.g. GFP and RNAi constructs) under the ubiquitous *actin* promoter once recombination is induced by the *esg* promoter. In this case, we observed GFP expression in all the clonal cells (Fig. S1A).

Because *Mira* expression is also strongly downregulated in PRC1-deficient cells according to our transcriptomic dataset (8.2-fold decrease; log₂FC = -3.03), using *Mira-Gal4* would not overcome this limitation: the reporter would likewise be lost due to the same identity change. This additional loss of a third progenitor marker (in addition to *esg* and Dl) further supports a profound disruption of progenitor identity in PRC1-deficient cells. Moreover, we performed an additional experiment to test the expression of Su(H), which is specifically expressed in enteroblasts, to be sure that TIICs are not EB. The results showed no expression of the reporter gene Su(H)-LacZ in tumors, confirming that TIICs are not EB. We added this experiment to the manuscript (Fig. S1B, new fifth row; lines 165 to 168).

Finally, we incorporated several other ISC markers found to be downregulated in our RNAseq experiment in addition to *mira* into Fig. 3B, confirming the loss of stem cell identity in TIIC tumors. Those genes are :

- *insc* – Pandey & Roy, Experimental Cell Research 2024
- *sox21a* – Doupé et al., PNAS 2018
- *zfh2* – Doupé et al., PNAS 2018

Regarding *piezo-Gal4*, we agree with Reviewer 2 that it has some overlapping expression patterns with Dl-Gal4, as already indicated in the main text (see lines 181-183). However, the classic EE-driver *ProsV1-Gal4* is also known to partially overlap with Dl (see Fig1A and He et al. 2018 Nature.555(7694):103-6). As for *rab3-GAL4*, it is expressed in EE, but not in EEps (see Li Y. et al., 2017, Sci. Rep. 7(1): 988 and Guo X. et al., 2024, Nature Communications 15:2656). Therefore, the *piezo-Gal4* driver remains the best specific driver for EEP.

***Reviewer 2 – Minor comment 1*:**

*Fig. 2 needs supplementary data to show if the esgF/O>Psc/Su(z)2^RNAi^ flies exhibited the tumor phenotype.*

Indeed, it is important to ensure that the *esgF/O>Psc/Su(z)2^RNAi^* flies used for the allografts exhibit the tumor phenotype. We presented these data in Supplementary Figure 1 (Fig. S1A, lower panels), which demonstrate that the *esgF/O>Psc/Su(z)2^RNAi^* flies (as well as *esgF/O>Psc/Su(z)2^DDN^*) exhibits the expected tumor phenotype. Additionally, we have updated the text to explicitly reference this control (lines 217-219).

***Reviewer 2 – Minor comment 2*:**

*Fig. 4A-B need the Dorsal^RNAi^ and Relish^RNAi^ alone controls.*

We performed these controls (*esg^ts^>dorsal^RNAi^* and *esg^ts^>Rel^RNAi^*), for which we observed no tumors, and have now added them to Fig. 4A (the last two rows of the panels) and modified the main text (lines 333-334).

***Reviewer 2 – Minor comment 3*:**

*Page 14, lines 343-344, the authors missed a conclusion sentence for these data. Are* *Zfh1 and Chimno not required for the PRC1^LOF^-tumor progression?*

This part of the text has been removed (see Reviewer 1 - Comment 3).
